# Fear of COVID-19 and secondary trauma: Moderating role of self-efficacy

**DOI:** 10.3389/fpsyg.2022.838451

**Published:** 2022-09-07

**Authors:** Yaling Li, Qamar Abbas, Shahjehan Manthar, Aftab Hameed, Zainab Asad

**Affiliations:** ^1^Mental Health Education and Counseling Center, Shenzhen Technology University, Shenzhen, China; ^2^Department of Management Science, Mohammad Ali Jinnah University, Karachi, Pakistan

**Keywords:** fear of COVID-19, secondary trauma, occupational self-efficacy, nurses, Smart PLS

## Abstract

COVID-19 has affected millions of people around the globe. People's mental health, especially those of nurses, has been primarily affected by the fear of this virus. More focus has been paid to vaccination and treatment of the virus, but less attestation has been given to addressing the mental health of people affected by the virus. Empirical studies show that different external factors are not easily manageable and controllable by the individual. This study preliminarily explores the connection between fear of COVID-19 and secondary traumatic stress in nurses. Further, it examines the moderating effects of occupational self-efficacy on the relationship between fear of COVID-19 and secondary traumatic stress. Data for the study was collected from the nurses of six large hospitals in Karachi, Pakistan. The final analysis was performed on 243 samples. Studies on COVID-19 suggest that increased occupational self-efficacy decreases fear and its impact. This study offers insights for managers to develop stress management programs and provide proper training and counseling sessions to the nurses to motivate them emotionally. Theoretically, this study broadens the understanding of the theory of emotions by using the pandemic as a stressor. Future studies may explore different roles of occupational self-efficacy and study its influential role in managing different kinds of emotions explained by the theory of emotions. Managers at the workplace could design different self-efficacy training for nurses to increase their self-motivation to fight different types of stress they face at the workplace.

## Background

COVID-19 is a global crisis that has ravaged the world economy and health sector and created fear and uncertainty among billions of people (Islam et al., 2021). The pandemic has badly affected all the countries in the world (Mumtaz, 2020) and led to long-term mental health issues (Fitzpatrick et al., 2020; Vagni et al., 2020; Pan et al., 2021), social isolation (Loades et al., 2020; Sepúlveda-Loyola et al., 2020; Zakeri et al., 2021), and shortage of protective equipment (Zakeri et al., 2021). Individual mental health has been severely affected during the pandemic (Khattak et al., 2020).

Medical studies hold that fear processing causes neuroinflammatory changes that lead to secondary trauma development (Sharma and Szaflarski, 2021). Fear of COVID-19 further results in multiple complications, including emotional arousal (Islam et al., 2021), fear of spreading the virus to other people (Vagni et al., 2020), psychological reactance (Akhtar et al., 2020), xenophobia (Mamun and Griffiths, 2020; Ahuja et al., 2021), intolerance and uncertainty (Satici et al., 2020b), depression, anxiety and stress (Bakioglu et al., 2021; Pan et al., 2021), and secondary trauma (Khattak et al., 2020). Emotions are always hard to regulate and patients who cannot regulate emotions like fear are always at risk of anxiety (Fosha, 2009) COVID-19 infected people of all ages and professions (Vagni et al., 2020). Still, nurses and paramedics experienced higher levels of trauma due to continuous work stress during the pandemic (Labrague and Santos, 2020). The extended workload was another central element that caused continuous stress and severe anxiety in nurses and paramedics (Kelly, 2020; Pfefferbaum and North, 2020). Secondary trauma was inevitable as they were engaged in an environment exposed to continuous distress (Elwood et al., 2011). Due to such stress and further stigmatization for working in infectious environments, nurses have been emotionally challenged to provide good healthcare services (Ramaci et al., 2020). Researchers have argued that mental health treatment is as necessary as the vaccine for the virus (Mamun and Griffiths, 2020). Mukhtar (2020) states that the situation in Pakistan is not much different from other countries and high levels of stress have been reported due to the COVID-19 crisis. The fear of the disease has also led to mental breakdowns and suicide attempts in Bangladesh, India, and the neighboring countries (Mamun and Griffiths, 2020). According to news reports, several suicide cases have been reported in Pakistan linked to the stress and fear created by COVID-19 (Goyal et al., 2020; Mamun and Ullah, 2020). Since the outbreak of COVID-19, post-traumatic syndrome disorders have increased in the public by seven percent (Torales et al., 2020). The National Institute of Health (NIH) reports indicate that nurses were the largest community affected by the fear of COVID-19 and related distress (Khattak et al., 2020). They were required to outperform, while at the same time there was comparatively less focus on the wellbeing of the involved staff (Kelly, 2020). Among nurses, the fear has increased mental stress due to their vulnerability. Unfortunately, the situation continues as the nursing community is still neglected and their plight unalleviated by relevant policymakers (Shahrour and Dardas, 2020). Healthcare organizations have paid less attention to the treatment or prevention of mental health issues such as fear and stress developed due to the virus (Mahmud and Talukder, 2020). Labrague and Santos (2020) indicated that nurses and paramedics were the frontline warriors in the pandemic and played a vital role in saving many lives; yet, they were the most highly affected community (Kelly, 2020).

Research has explored different relationships to reduce COVID-19 stress and fear in the workplace. Some evaluated leadership support to moderate fear of COVID-19 and stress (Khattak et al., 2020); others found that the role of media also regulated the relationship between fear of COVID-19 and public panic (Xu and Sattar, 2020). Most of the factors and attributes examined as moderators or mediators are external factors, which are not linked directly to the individual. Daniali and Flaten (2021) also recommended exploring the intrinsic personal factors that may respond to treatment. This study, therefore, examines the effects of fear of COVID-19 on secondary trauma in nurses and paramedics in the hospitals in Karachi. It also investigates the moderating effects of occupational self-efficacy between the coupled relationship of fear of COVID-19 and secondary traumatic stress.

## Theoretical background

The theoretical support for this study is derived from the theory of emotions by James (1884); Lang (1994). The theory proposes that emotion can stimulate the senses and lead to bodily changes, An apprehended object, turns into an object emotionally felt (Cannon, 1927). The theory of emotions offers the view that somatic symptoms developed from emotion may cost much to individuals, as their quality of life is reduced and health systems are affected (Barlow, 2004). The theory explains that impressions of the world enrich our views, but our emotions stimulate them in different situations such as anger, happiness, and fear (Dana, 1921; Cannon, 1927). Emotions synchronize changes in all subsystems of the body that arise from an external or internal stimulus (Scherer, 2005). Dana (1921) noted that most of the emotions fall under love and fear, and feelings that need retreat are related to fear (Harlow and Stagner, 1933). Barlow (2004) perceived that emotions containing fear would increase anxiety, one of the core arousal symptoms leading to secondary trauma (Metzger et al., 2004). The fear of COVID-19 has led to somatic symptoms, distress, and anxiety (Ahorsu et al., 2020; Satici et al., 2020a). This study, therefore, has used the theoretical framework of the theory of emotion to examine the occurrence of fear as stimuli for secondary traumatic stress.

### Fear of COVID-19 and secondary trauma stress

Bourke (2003) noted that the definition of fear changes based on the situation. It is commonly defined as a reaction to perceived or imagined threats and the anticipation of the risk of possible attacks (Gullone, 2000; Bourke, 2003). Fear is an emotion (Ledoux, 1995), and any form of broader fear can cause a loss of overall emotions, and may also lead to panic disorder (Berg et al., 1998). COVID-19 is perceived as a severe threat to people's lives and social wellbeing (Abdullah, 2020), and a dominant source of stress, anxiety, and fear worldwide (Reznik et al., 2020). Secondary trauma is characterized as a tremor in the social structure caused by an inadequate response to an initial disaster or incident (Gill, 2007). Vagni et al. (2020) noted that COVID-19 fear relates to the risk involving transmission of the virus to different people or loved ones, which causes more distress. COVID-19 consists of many stressors and develops a traumatic situation for different groups of people for different reasons (Kira et al., 2020). Usually, the reactions to stress and re-experiencing the trauma triggers secondary traumatic stress (Khattak et al., 2020). Countries have different responses to mental health issues caused by the virus. The US declared COVID-19 a crisis in mental health (Fitzpatrick et al., 2020). The risk of losing a life is the major contributor to the development of secondary trauma (Vagni et al., 2020).

Ornell et al. (2020) stated that people whose mental health is affected are more numerous than the people infected by the virus. Initial studies in Pakistan also indicated that exposure to the novel coronavirus has led to secondary trauma (Amin, 2020). Generally, the healthcare industry and paramedics were exposed to and affected more by this paramedic (Arpacioglu et al., 2020; Khattak et al., 2020). As they have extended contact with patients, not only stress levels and development of secondary trauma is higher in people working in the healthcare sector, but they also face severe psychological and mental challenges (Khattak et al., 2020; Vagni et al., 2020). It is further evident from research that nurses and paramedics come across mental health issues involving stress, anxiety, and psychological disorders (Labrague and Santos, 2020). Yildirim and Güler (2020) observed that people exposed to this virus were more sensitive to feeling stress or experiencing trauma.

### Moderating effect of occupational self-efficacy

According to Bandura (1986), self-efficacy refers to individual abilities and beliefs that help execute a different course of action for the attainment of designated performances, and occupational self-efficacy (OCSE) refers to the execution of self-efficacy beliefs to attain desired goals in the work environment (Schyns and von Collani, 2002). OCSE is viewed as an essential organizational resource and enables employees to adapt to different work situations and behavior (Rigotti et al., 2008; Pisanti et al., 2015). OCSE also explains several outcomes associated with individual beliefs (Cetin and Askin, 2018) and transforms individuals' capabilities. Maggiori et al. (2016) believed that individual beliefs have a role, but job requirements and different work dimension changes the level of perceived self-efficacy in individuals. Gecas (1989) described that OCSE shapes intellectual flexibility, and affects self-direction, and different aspects of one's personality. It carries healthy behaviors including self-esteem, optimism, and the ability to socially express oneself, which help individuals in their professional wellbeing (Maggiori et al., 2016). It enables self-motivation in workers to perform better in their tasks (Paggi and Jopp, 2015). Different authors studied different relationships to deal with stress and trauma caused by the fear of COVID-19 at the workplace. Occupational self-efficacy has been studied with different variables across different disciplines. It significantly influences human resource development and work engagement (Chaudhary et al., 2012), personal traits, leadership perceptions (Felfe and Schyns, 2006), job strain, job satisfaction (Maggiori et al., 2016), psychological climate, job security (Tomas et al., 2019) and career assessment (Betz and Hackett, 1997).

**Figure 1:**
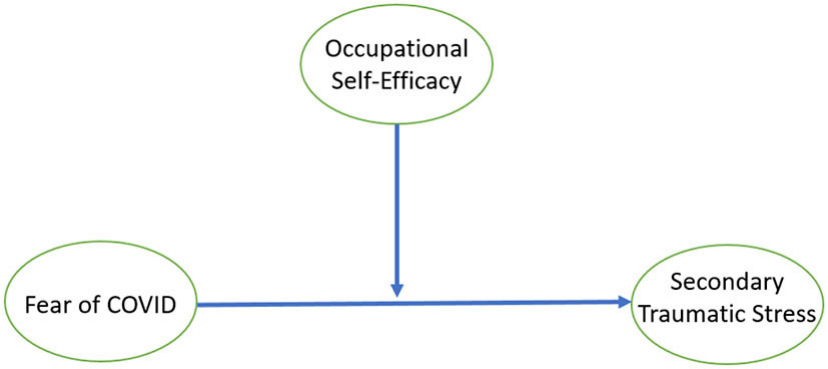
Conceptual framework.

This study used OCSE as a moderator because several pieces of empirical evidence are available that show that occupational self-efficacy has a significant relationship with secondary traumatic stress (Sun et al., 2020). Bandura's cognitive social theory holds that exercising control over stressful situations needs high self-efficacy levels, and occupational self-efficacy is a better choice to moderate stressors (Grau et al., 2001). The theory of self-efficacy also supports the narrative that individual motivation can help one to attain a specific goal during complex challenges. Cepale et al. (2021) noted that self-efficacy provides solutions for the workplace socialization process and helps to reduce intentions to quit due to stress employees face at work. Sometimes internal negative factors create stress and fear; training and organizational support to boost self-efficacy can help in this situation (Thornberry et al., 2020). Islam et al. (2021) also recommended conducting a study on behavioral outcomes and traits, and to explore the intrinsic personal factors that may be useful in tackling the fear of COVID-19 (Daniali and Flaten, 2021). This study, therefore, undertakes to examine the moderating role of occupational self-efficacy in the relationship between fear of COVID-19 19 and secondary traumatic stress. The proposed research model has been shown in Figure 1.

## Study aim and hypothesis

Considering the above empirical evidence, this study examines the influence of fear of COVID-19 on secondary traumatic stress in nurses in healthcare settings in Karachi, Pakistan. Previous studies have explored the external links in the relationship between fear of COVID-19 and secondary traumatic stress. This study aims to examine the role of any such variable that stems from an individual's personality. Assuming that the role of occupational self-efficacy (as a moderator that weakens the relationship between fear of COVID-19 and secondary traumatic stress), the following hypotheses have been identified:

- ***H***_**1**_***:*** *Fear of COVID-19 has a significant influence on secondary traumatic stress*
- ***H***_**2**_***:*** *Occupational self-efficacy significantly moderates between fear of COVID-19 and secondary traumatic stress*.

## Methods

### Design

The deductive approach was used in this study to explain different causal relationships between variables. As suggested by Hair et al. (2013) and adapted by Zhao et al. (2020), the minimum sample size was selected based on a power analysis by assessing the more significant number of predictors pointing toward the dependent variable. The suggested size with two predictors was 52 with a statistical power of 80%, an R-squared value of 0.25, and a 5% significance level.

### Measures

The fear of COVID-19 scale (FCS) developed by Ahorsu et al. (2020) was used in this study, and all seven items of the construct were adopted in this research. A five-point Likert scale was adopted where one “Strongly Disagree” to five “Strongly Agree” was used to measure the responses to the fear of COVID-19. The scale developed by Ahorsu et al. (2020) has already been validated in different contexts and countries (Alyami et al., 2020; Martínez-Lorca et al., 2020; Perz et al., 2020; Reznik et al., 2020; Satici et al., 2020a). Pakpour et al. (2020) noted that the FCS by Ahorsu et al. (2020) has solid psychometric qualities.

The measure of secondary traumatic stress scale was taken from (Bride et al., 2004), and all 17 items were adopted in this research. To assess the validity, different earlier studies were referred to check the contexts in which this construct was previously used. The scale was found valid in different contexts of different studies conducted in China (Zhong et al., 2021), the US (Armes et al., 2020), Turkey (Arpacioglu et al., 2020), and 14 different studies using the same scale have been referred in a cross-sectional analysis conducted by Molnar et al. (2020).

A shorter version of the OCSE scale previously used by Rigotti et al. (2008) was applied in this study to assess the moderating effect of occupational self-efficacy on secondary trauma. The original scale had 20 items and it was developed by Schyns and von Collani (2002). The first shorter version of this construct was found reliable and validated on a German sample. A five-point Likert scale from one “strongly disagree” and five “strongly agree” was used in this study to examine the responses statistically. Different research studies have also translated and validated the shorter version of the OCSE scale (Hirschi and Jaensch, 2015; Clauss et al., 2020; Weber et al., 2020).

### Participants and procedure

Data was collected from the nursing staff from September to November 2020. The study's objectives were discussed with the administrative wing/divisions of hospitals, and permission was requested to collect data from COVID-19 nursing units. The administration requested us to avoid direct contact with COVID-19 nursing staff without appropriate protective measures. About 320 questionnaires were distributed to nurses and nursing assistants at six large hospitals in Karachi. Each hospital nominated a person to distribute the questionnaires to the concerned nursing staff, and ample time was provided to complete the responses, preferably during their holidays and work off. Of the distributed questionnaires, 298 were received from the focal persons in a sealed and identified manner, and finally, 243 questionnaires with complete responses were processed for final analysis. Table 1 shows the demographic representation of the participants.

**Table 1:** Demographic representation of the respondents.

**Gender**	**Nos**.	**Percentage**
Male	130	53.50%
Female	113	46.50%
**Age**		
19 or less	03	1.23%
20–29	79	32.51%
30–40	151	62.13%
41–50	05	2.05%
51–60	04	1.65%
More than 60	01	0.42%
**Qualification**		
Matric	28	11.52
Intermediate	83	34.15
16 years of education	89	36.63
18 years of education	39	16.04
PhD	04	1.65
**Designation/position**		
Nurses	170	69.96%
Nursing assistants	73	30.04%

*n* = 243

### Common method biases

To identify the common method bias (CMB), this study followed the procedure suggested by Podsakoff et al. (2011) using proximal and psychological separation of constructs. Later, full collinearity was also checked statistically to determine the data bias as Kock (2015) and Kock and Lynn (2012) suggested. The results shown in Table 2 indicate that the value of variance inflation factor (VIF) of all variables was <3.3, which holds that common method bias was not a severe problem for this study.

**Table 2:** Full collinearity analysis.

	**FoC**	**OCSE**	**STS**
VIF	1.138	1.138	1.178

FoC, fear of COVID, OCSE, occupational self-efficacy; STS, secondary traumatic stress.

### SEM results

**Figure 2:**
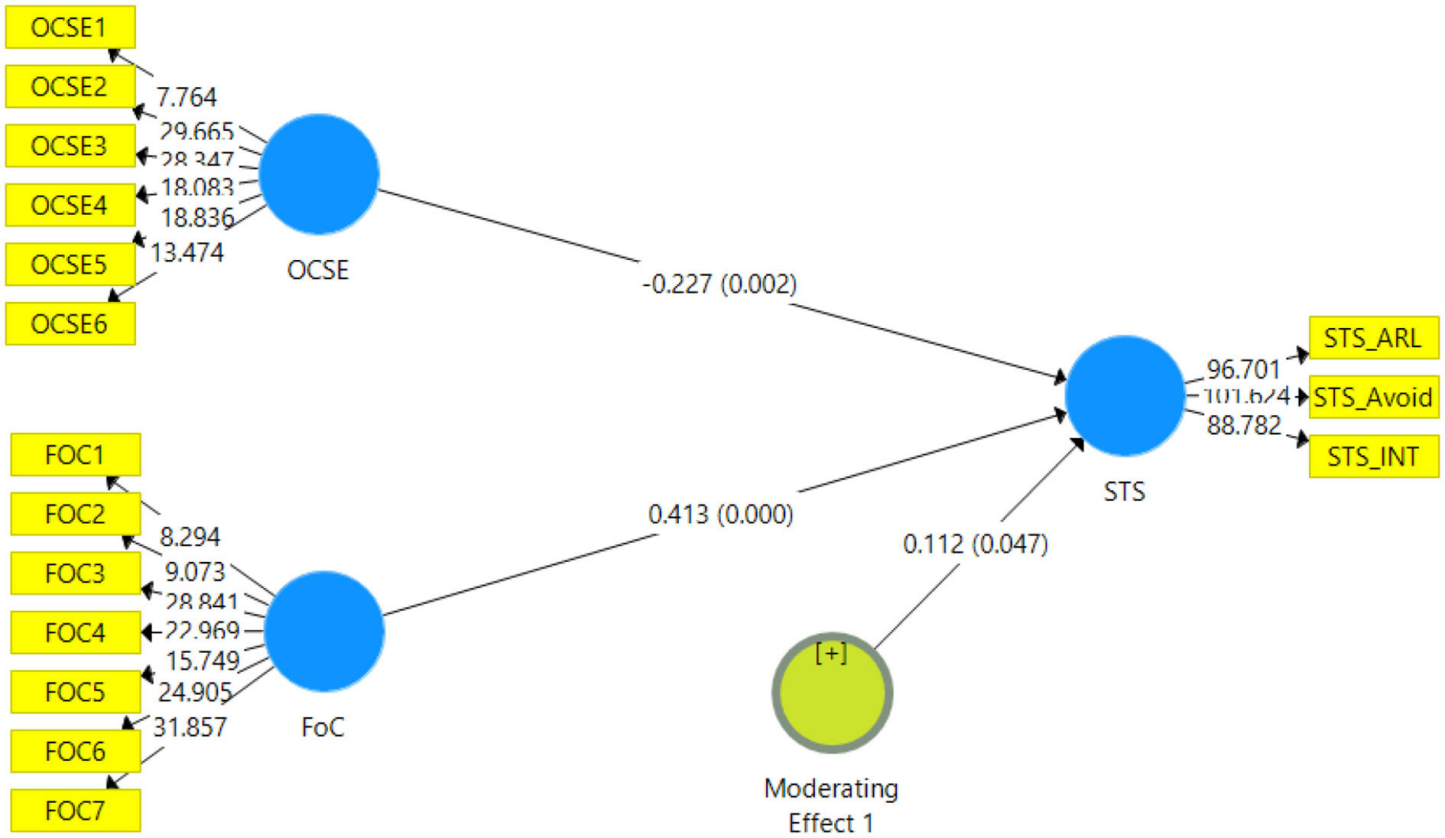
Estimated model.

The structural model was assessed using Smart PLS 5000 resample bootstrapping procedure suggested by Ramayah et al. (2020). The estimated model is displayed in Figure 2. Secondary traumatic stress has three dimensions - arousal, avoidance, and intrusion, estimated in higher order. Avoidance had seven items whereas arousal and intrusion had five items each. Occupational self-efficacy had six items and fear of COVID-19 had a total of seven items.

Results are shown in Table 3, fear of COVID-19 significantly impacted secondary traumatic stress (β = 0.166, *p* < 0.01), supporting H1 of this study. H_2_ of this study was also supported, as the results indicated the impact of fear of COVID-19 was reduced by 31% with occupational self-efficacy moderating between the fear of COVID-19 and secondary traumatic stress (β = 0.166, *p* < 0.01).

**Table 3:** Structural model/ Hypothesis testing results.

**Hypothesis**	**Values**	**Decision**
FoCovid -> STS (H_1_)	0.413^**^	Accepted
Moderating Effect 1 -> STS (H_2_)	0.112^*^	Accepted

*, ** Significant at 5 and 1%, respectively.

## Discussion

This research aimed to examine the influence of fear of COVID-19 on nurses' psychological and mental health, i.e., secondary traumatic stress. This study also explained a mechanism of how the intensity of fear was decreased in nursing staff with the support of occupational self-efficacy as a moderator. The study results found that the relationship between fear of COVID-19 with secondary traumatic stress in nursing hospitals in Karachi was statistically significant and positive (β = 0.413, *p* < 0.01). Since nurses were exposed more to COVID-19 patients, the risk of infection and feelings of fear may be found to be more in their case (Labrague and Santos, 2020). The National Institute of Health (NIH) had also revealed in its reports that nurses were the largest community suffering from the fear of COVID, and its distress (Khattak et al., 2020). Pakistan has a weak occupational safety system, and the risk factor of psychological distress is more common among nurses (Amin, 2020). Results of this study have validated different arguments of other researchers who found that fear of COVID-19 is a stressor that affects the mental and psychological health of nurses and paramedics. Since nurses are on the frontline of the pandemic (Abdo et al., 2021), they should be equipped with the latest information and support so that their fear and stress level may be reduced (Labrague and Santos, 2020). A psychological response is necessary to cope with this fear, but no clinical efforts have been made so far to deal with this fear (Ahorsu et al., 2020). Necessary measures must be taken at the organizational level (Sawhney et al., 2020). Healthcare organizations should have a well-designed protocol to deal with all kinds of disasters (Sawhney et al., 2020), and care for the mental health of nurses should be taken into consideration (Mamun and Griffiths, 2020). Most of the research recommendations support urgent care and proper mental health strategies (Torales et al., 2020). The most common suggestions received from the researchers to deal with trauma, highlight the need to reduce the workload (Elwood et al., 2011).

At this moment, it seems unclear when the pandemic might end; but preventive measures and empirical knowledge may help cope with this situation (Mukhtar, 2020). A specific vaccine was not available for the COVID-19 virus (Ornell et al., 2020; Zhai et al., 2020), and no specific time can be predicted to end this pandemic (Yousaf et al., 2020). Even though social distancing and other safety measures provide safety (McKay et al., 2020), mental health is likely to be affected by living in isolation (Mukhtar, 2020). Academic researchers are contributing, so that the impact of the fear of this virus may be minimized. Different empirical studies have tested different relationships to check how different variables affect the relationship between fear of COVID-19 and secondary traumatic stress (Khattak et al., 2020; Xu and Sattar, 2020). This study has examined the moderating effect of occupational self-efficacy in a relationship between fear of COVID-19 and secondary traumatic stress. The study results indicated that the relationship became weak when the OCSE moderated the relationship. Few studies indicate similar results of occupational self-efficacy moderating the relationship between different occupational stressors and secondary traumatic stress (Schaubroeck and Merritt, 1997; Grau et al., 2001). The results lead to the understanding that OCSE may curtail the effect and severity of the COVID-19 fear and secondary trauma. Positive support from the organization or peers may reduce the stress level of nurses (Labrague and Santos, 2020). However, a generalized view cannot be established that fear of COVID-19 is directly associated with secondary trauma, but more factors influence and strengthen its effect. For instance, all the samples may not possess occupational self-efficacy. Still, the results of the moderating effect of OCSE indicated that the strength of fear of COVID-19 was reduced from 43 to 11%.

## Implications

COVID-19 is a new virus and has received worldwide attention from researchers and practitioners. Even though a lot of the work has already been done, more is required from the academic community. This study draws two significant theoretical implications: First, it provides theoretical support to the literature on fear of COVID-19, secondary traumatic stress, and occupational self-efficacy. Secondly, it provides extended support to the existing theory of emotions by adding and testing a new variable i.e., fear of COVID-19 on stress, and proving that a pandemic can cause a negative stimulus, affect emotions, and lead to bodily changes. Theoretically, this study broadens the understanding of the theory of emotions by using the pandemic as a stressor. Future studies may explore different roles of occupational self-efficacy and study its effective role in managing different kinds of emotions explained by the theory of emotions.

It is also inferred from the results that organizations, especially healthcare institutions, should provide vital support to nurses to cope with occupational stress. The study draws that a comprehensive stress management system should be developed by organizations in the health sector and be responsible for providing mental and psychological support to the workers, along with offering training related to COVID-19, counseling sessions, and other programs. Psychological consultants should be engaged to check and counsel nurses and encourage them to face these challenges. HR departments of relevant hospitals also need to devise proper training for nurses and paramedics focusing on increasing their self-efficacy and self-motivation.

## Limitations and future research directions

This study has some limitations; accessibility was the main issue we faced during data collection. The hospitals had busy working schedules with an increased number of patients. The availability of all nurses was difficult and the actual respondents were therefore low in numbers compared to the total strength of nurses at the hospital. Available time was limited, which restricted this study to three variables only. The non-availability of proper funding limited us to conducting the study at a basic level. Future studies could examine more variables and add age or gender as categorical variables to check and compare secondary traumatic stress effects between genders or younger and older adults. This study introduced and significantly examined an anti-stressor to weaken the effect of fear of COVID-19, which will help researchers to explore more variables that can reduce occupational stress. Further, the model explained in this study can be extended by using a few more variables, such as organizational support and flexible working hours; and moderators of perceived organizational support as mediators. Future models could also use occupational self-efficacy as a mediator to check their direct and indirect effects on secondary trauma and other stressors. Lastly, this is a cross-sectional study, and future studies may conduct a longitudinal study to test and validate the changes that occur over time.

## Conclusions

Our study examined the different effects of COVID-19 fear on secondary trauma. It explored the fear of COVID-19 that caused distress and increased the mental stress of nurses and paramedics in selected large hospitals in Karachi, Pakistan. The study further examined the moderating effect of occupational self-efficacy and examined how it can help reduce fear of COVID-19 in healthcare professionals. The results established that fear of COVID-19 poses serious issues for the mental and psychological health of nurses and paramedics in Pakistan. To address the problem, this study found support from empirical studies and used occupational self-efficacy as a moderator to determine, if the intensity of the fear could be reduced. The results of the study suggest that OSCE is a good anti-stressor and can reduce secondary traumatic stress. The study also highlights the need for remedial measures to address the mental health issues of nurses related to the fear of COVID-19. The study's external validity may vary in different contexts and settings, and this study has provided adequate information that “fear” as an emotion can be reduced by the interplay of self-motivation and occupational self-efficacy. The impact created by occupational self-efficacy may differ in other settings for many reasons, such as healthcare settings that offer proper training on self-motivation, counseling of their nurses, and counter work-stressors that can turn the relationship negative between any kind of fear and secondary traumatic stress. Likewise, in settings where no proper care is provided to nurses and their work output is the only goal of the institution, self-efficacy may not be as effective as in a positive work environment.

## What is already known about the topic

- Different roles of fear of COVID-19 have been discussed previously, including fear of COVID-19 and IPO performance, fear of COVID-19 and absenteeism, fear of COVID-19 and anxiety, and fear of COVID-19 and oral health perceptions.
- The relationship between fear of COVID-19 and depression and secondary traumatic stress has been fairly discussed.
- Factors moderating between fear of COVID-19 and secondary trauma have also been studied, but the studied moderators have no direct link with respondents or subjects. The variables such as organizational support, leadership support, and work environment are some of the variables explored in different studies, and none of them are directly linked to individual personality.

## What this paper adds

- This paper discusses a new moderator which has a direct association with individual personality. This variable has a role in weakening the relationship between fear of COVID-19 and secondary traumatic stress.
- This paper also explores a link to how a pandemic or disaster can affect emotions which further lead to secondary traumatic stress.
- Further discussions on the link between fear, secondary trauma, and occupational self-efficacy have also provided significant support to the literature on the theory of emotions.

## Data availability statement

The raw data supporting the conclusions of this article will be made available by the authors, without undue reservation.

## Ethics statement

The studies involving human participants were reviewed and approved by Ethical Committee of Mohammad Ali Jinnah University. Written informed consent for participation was not required for this study in accordance with the national legislation and the institutional requirements.

## Author contributions

YL: supervision, fund acquisition, and project administration. QA: conceptualization, software, and formal analysis. QA, SM, and ZA: methodology. ZA and SM: data collection. SM: writing original draft preparation. AH: writing—review and editing and validation. All authors contributed and approved the final version of the article.

## Funding

This work was supported by Shenzhen 2021 Philosophy and Social Science Planning Project (No. SZ2021B036), the 14th Five-Year Plan Education Research Project of Guangdong Education Association in 2021 (No. GDESH14006), and Shenzhen Education Society's 14th Five-Year Plan 2021 Educational Research Project (No. ZD2021002).

## Conflict of interest

The authors declare that the research was conducted in the absence of any commercial or financial relationships that could be construed as a potential conflict of interest.

## Publisher's note

All claims expressed in this article are solely those of the authors and do not necessarily represent those of their affiliated organizations, or those of the publisher, the editors and the reviewers. Any product that may be evaluated in this article, or claim that may be made by its manufacturer, is not guaranteed or endorsed by the publisher.
